# Supplementary material for: MechRAG: a multimodal large language model for mechanical engineering
Source: Commun Eng. 2025 Nov 11;4:187. doi: 10.1038/s44172-025-00517-z (PMC12606259; doi:10.1038/s44172-025-00517-z)
Supplement: Supplementary file 2 — Supplementary Information [file 44172_2025_517_MOESM2_ESM.pdf]

## Supplementary information

```
image_prompt = ChatPromptTemplate.from_messages([
    ("system", """You are a mechanical engineer with expertise in manufacturing processes.
    You will be provided with an image of the target mechanical part,
    along with images and manufacutring process descriptions of similar parts to the target part.
    Your job is to use the images and manufacturing processes of the similar parts to describe the target part.
    """),

    ("user", [
        {"type": "text", "text": """The tartget mechanical part is shown in the image."""},
        {"type": "image_url", "image_url": "data:image/jpeg;base64,{image_query}"},
        {"type": "text", "text": """There are images and manufacturing processes of similar mechanical parts to the target part."""},
        {"type": "image_url", "image_url": "data:image/jpeg;base64,{retrieved_info_images_0}"},
        {"type": "image_url", "image_url": "data:image/jpeg;base64,{retrieved_info_images_1}"},
        {"type": "image_url", "image_url": "data:image/jpeg;base64,{retrieved_info_images_2}"},
        {"type": "text", "text": """they are individually manufacuted with the processes of"""},
        {"type": "text", "text": "{retrieved_info_texts}"},
        {"type": "text", "text": """Respond with a concise answer stating the manufacutring process used to produce the target part"""},
    ]),
])
```

**Supplementary Figure 1.** An example of simple prompting used in the experiments

```
image_prompt = ChatPromptTemplate.from_messages([
    ("system", """You are a mechanical engineer with expertise in manufacturing processes.
    You will be provided with an image of the target mechanical part,
    along with images and manufacutring process descriptions of similar parts to the target part.
    Your job is to use the images and manufacturing processes of the similar parts to describe the target part.
    """),

    ("user", [
        {"type": "text", "text": """The tartget mechanical part is shown in the image."""},
        {"type": "image_url", "image_url": "data:image/jpeg;base64,{image_query}"},
        {"type": "text", "text": """There are images and manufacturing processes of similar mechanical parts to the target part."""},
        {"type": "image_url", "image_url": "data:image/jpeg;base64,{retrieved_info_images_0}"},
        {"type": "image_url", "image_url": "data:image/jpeg;base64,{retrieved_info_images_1}"},
        {"type": "image_url", "image_url": "data:image/jpeg;base64,{retrieved_info_images_2}"},
        {"type": "text", "text": """they are individually manufacuted with the processes of"""},
        {"type": "text", "text": "{retrieved_info_texts}"},
        {"type": "text", "text": """Analyse the target part and the similar parts following the process:
        1. Step 1: Visual inspection for every part:
            a. Observe the shape in the image, focus on the geometric features.
        2. Step 2: Manufacturing method analysis for every part:
            a. Identify features that strongly indicate specific manufacutring methods.
            b. Also list other manufacutring methods possiblely related to the part based on the observations.
        3. Step 3: Comparison between the target part and the similar parts:
            a. Compare the target part and the similar parts based on the outputs from Step 1 and Step 2.
               If the target part and the similar parts are very alike, then the manufacturing process description of the target part should be
               similar to the ones of the similar parts.
               If the target part and the similar parts are not alike, then describe the manufacturing process of the target part independently.
        4. Step 4: Summary of the conclusion:
            a. If from the previous steps the target part is likely to be manufactured by a sheet metal/fabricated/forged/turned manufacturing process,
               go ahead and output the conclusion.
               If from the previous steps the target part is unlikely to be manufactured by a sheet metal/fabricated/forged/turned manufacturing process,
               categorise it as being manufactured by other manufacturing processes and output the conclusion.

        """},
        {"type": "text", "text": """Respond with a concise answer stating the manufacutring process used to produce the target part"""},
    ]),
])
```

**Supplementary Figure 2.** An example of CoT (Chain-of-Thought) used in the experiments

```
method_mapping = {
    'fabricated': ['fabricated', 'fabrication', 'weld', 'welding', 'glue', 'gluing', 'adhesive'],
    'forged': ['forged', 'forging', 'hammer', 'beat'],
    'sheet metal': ['bend', 'bending', 'sheet', 'plate', 'punch', 'press', 'cut', 'stamped', 'shearing'],
    'turned': ['turned', 'turn', 'turning', 'rotational', 'spinning', 'rotary', 'rotating'],
    'other': ['other', 'machined', 'machining', 'CNC machining', 'injection', 'mold', 'mould', 'molded',
            'precision casting', 'casting', 'sand', 'milling', 'additive', 'additive manufacturing', '3D print',
            'extrusion', 'extruded','tube bending']
}
```

**Supplementary Figure 3.** The method for the manufacturing method identification task

**Supplementary Table 1.** Performances of different MechRAG configurations regarding different manufacturing methods

| Configuration | Manufacturing method | Accuracy | Precision | F1 score |
|---|---|---|---|---|
| GPT+CLIP | Fabricated | 84.31% | 74.14% | 78.90% |
| | Forged | 84.31% | 74.14% | 78.90% |
| | Other | 47.06% | 92.31% | 62.34% |
| | Sheet metal | 90.20% | 79.31% | 84.40% |
| | Turned | 98.04% | 90.91% | 94.34% |
| GPT+ViLT | Fabricated | 68.63% | 66.04% | 67.31% |
| | Forged | 86.27% | 67.69% | 75.86% |
| | Other | 39.22% | 86.96% | 54.05% |
| | Sheet metal | 80.39% | 67.21% | 73.21% |
| | Turned | 96.08% | 92.45% | 94.23% |
| GPT+image summary and text embedding | Fabricated | 52.94% | 22.50% | 52.94% |
| | Forged | 37.25% | 65.52% | 37.25% |
| | Other | 5.88% | 50.00% | 5.88% |
| | Sheet metal | 56.86% | 38.16% | 56.86% |
| | Turned | 41.18% | 87.50% | 41.18% |
| GPT+1% data | Fabricated | 50.98% | 49.06% | 50.00% |
| | Forged | 58.82% | 81.08% | 68.18% |
| | Other | 37.25% | 67.86% | 48.10% |
| | Sheet metal | 70.59% | 51.43% | 59.50% |
| | Turned | 100.00% | 76.12% | 86.44% |
| GPT+2% data | Fabricated | 58.82% | 65.22% | 61.86% |
| | Forged | 64.71% | 71.74% | 68.04% |
| | Other | 33.33% | 80.95% | 47.22% |
| | Sheet metal | 86.27% | 57.89% | 69.29% |
| | Turned | 98.04% | 75.76% | 85.47% |
| GPT+5% data | Fabricated | 80.39% | 73.21% | 76.64% |
| | Forged | 78.43% | 70.18% | 74.07% |
| | Other | 33.33% | 89.47% | 48.57% |
| | Sheet metal | 86.27% | 69.84% | 77.19% |
| | Turned | 98.04% | 83.33% | 90.09% |
| GPT+20% data | Fabricated | 82.35% | 85.71% | 84.00% |
| | Forged | 88.24% | 86.54% | 87.38% |
| | Other | 62.75% | 96.97% | 76.19% |
| | Sheet metal | 96.08% | 80.33% | 87.50% |
| | Turned | 100.00% | 85.00% | 91.89% |
| Claude | Fabricated | 96.08% | 56.98% | 96.08% |
| | Forged | 80.39% | 73.21% | 80.39% |
| | Other | 31.37% | 94.12% | 31.37% |
| | Sheet metal | 54.90% | 80.00% | 54.90% |
| | Turned | 98.04% | 83.33% | 98.04% |
| GPT+No RAG | Fabricated | 52.94% | 40.30% | 45.76% |

| | | | | |
|---|---|---|---|---|
| | Forged | 33.33% | 73.91% | 45.95% |
| | Other | 64.71% | 49.25% | 55.93% |
| | Sheet metal | 23.53% | 27.27% | 25.26% |
| | Turned | 86.27% | 86.27% | 86.27% |
| Claude+No RAG | Fabricated | 7.84% | 40.00% | 13.11% |
| | Forged | 21.57% | 100.00% | 35.48% |
| | Other | 58.82% | 41.10% | 48.39% |
| | Sheet metal | 78.43% | 41.67% | 54.42% |
| | Turned | 92.16% | 72.31% | 81.03% |
| Claude+CoT | Fabricated | 80.39% | 100.00% | 80.39% |
| | Forged | 66.67% | 100.00% | 66.67% |
| | Other | 49.02% | 100.00% | 49.02% |
| | Sheet metal | 98.04% | 100.00% | 98.04% |
| | Turned | 88.24% | 100.00% | 88.24% |
| GPT+CoT | Fabricated | 90.20% | 100.00% | 94.85% |
| | Forged | 86.27% | 100.00% | 92.63% |
| | Other | 70.59% | 100.00% | 82.76% |
| | Sheet metal | 100.00% | 100.00% | 100.00% |
| | Turned | 100.00% | 100.00% | 100.00% |

**Supplementary Table 2.** A simple example of the information contained in Dataset 2 for a single model

| | |
|---|---|
| Explicit information | The volume of the model_00210041 is 291396.544 cubic MM.<br>The surface area of the model_00210041 is 28739.346 square MM.<br>The volume of this model_00210041 bounding box is 333496.254 cubic MM.<br>The variance of the Gaussian curvature of this model_00210041 is 1.061.<br>The variance of the Mean curvature of this model_00210041 is 0.000.<br>The flat plane of this model_00210041 occupies 100.000%.<br>The curved surface of this model_00210041 occupies 0.000%.<br>The straight edge of this model_00210041 occupies 100.000%.<br>The curved edge of this model_00210041 occupies 0.000%.<br>This model_00210041 has no symmetry.<br>The axis of symmetry of this model_00210041 is None.<br>Total number of round holes of this model_00210041 is 0. |
| Modelling history | model_00210041 creates a sketch named Sketch 1 with the following features:<br>model_00210041 creates a line ending at (0.0254, -0.09867195).<br>model_00210041 creates a line ending at (0.0254, 0.0).<br>model_00210041 creates a line ending at (0.0254, 0.00292805).<br>model_00210041 creates a line ending at (0.0048512, 0.01785811).<br>model_00210041 creates a line ending at (0.0048512, 0.03055811).<br>model_00210041 creates a line ending at (0.0, 0.03055811).<br>model_00210041 creates a line ending at (-0.0048512, 0.03055811).<br>model_00210041 creates a line ending at (-0.0048512, 0.01785811). |

| | |
|---|---|
| | model_00210041 creates a line ending at (-0.0048512, 0.01785811).<br>model_00210041 creates a line ending at (-0.0254, 0.00292805).<br>model_00210041 performs an extrude operation named Extrude 1 with the following parameters:<br>model_00210041 sets the direction to (0, 0, 0),<br>model_00210041 sets the origin to (0, 0, 0),<br>model_00210041 sets the scale to 1,<br>model_00210041 sets the extent one to 0.025400000000000002,<br>model_00210041 sets the extent two to 0.025400000000000002,<br>model_00210041 sets the operation to NewBodyFeatureOperation,<br>model_00210041 sets the extent type to TwoSidesFeatureExtentType. |
